# A model-driven approach for fast modeling of three-dimensional laser point cloud in large substation

**DOI:** 10.1038/s41598-023-42401-w

**Published:** 2023-09-26

**Authors:** Ruiheng Li, Lu Gan, Yang Liu, Yi Di, Chao Wang

**Affiliations:** 1 School of Information Engineering, Hubei University of Economics, Wuhan, 430205 China; 2 State Key Laboratory of Power Transmission Equipment and System Security and New Technology, Chongqing, 400044 China; 3 School of Electrical Engineering, Chongqing University, Chongqing, 400044 China; 4 School of Computer and Information Science, Chongqing Normal University, Chongqing, 401331 China

**Keywords:** Power stations, Civil engineering

## Abstract

Using point cloud to reconstruct the 3D model of a substation is crucial for smart grid operation. Its main objective is to swiftly capture equipment point cloud data and align each device’s model within the large and noisy point cloud scene of the substation. However, substation reconstruction needs improvement due to the low efficiency of traditional noise-resistant clustering methods and challenges in accurately classifying similar-looking electrical equipment. This paper proposes an automatic modeling framework for large-scale substation point cloud scenes. Firstly, we reduce the substation scene’s dimensionality to improve clustering efficiency and establish relationships between data dimensions using a re-clustering algorithm. Next, a neural network is developed to identify various device types within clusters, even with limited subdivisions. Finally, a model library is employed to register standard models onto the target device’s point cloud, obtaining device types and orientations. Real substation data processing demonstrates the ability to rapidly extract devices from complex and noisy point cloud scenes, effectively avoiding missegmentation issues. The automatic modeling approach achieves a precise substation calculation rate of 92.86%.

## Introduction

With the rapid advancement of smart grids, the demand for rapid 3D scene modeling in substations is on the rise. In comparison to traditional CAD modeling and tilt photography methods, the 3D laser scanning modeling approach stands out as a method that places existing models into simulation scenarios using device point clouds. This method offers high accuracy and excellent scene integrity, effectively meeting various high-precision modeling demands^1,2^.

At present, the modeling process relies mostly on manual processing. First, the point clouds of equipment in a point cloud scene are divided; next, the specific types of equipment are judged manually based on real-world experience; and then, the corresponding equipment model is found in the model library and manually dragged into the scene. The whole process revolves around two critical issues: (1) extracting the target equipment point clouds and (2) identifying the types of equipment represented by the point clouds.

Many useful point cloud clustering algorithms for extracting a single target from a point cloud scene have been published. These methods are mainly divided into two categories: clustering algorithms based on distance information and clustering algorithms based on density information^3–5^. However, the actual effects of these two types of algorithms are affected by noise^6–8^.

For clustering algorithms based on distance information, the clustering results depend mainly on the distance threshold set in advance. The prerequisite for obtaining a better clustering effect is that the interval of each target cluster has a fixed rule^9,10^. When the point cloud scene contains considerable noise, the noise will connect the target clusters, making it difficult to determine the distance threshold^3^.

Clustering algorithms based on density information can address the trouble caused by noise to a certain extent. The main principle is to judge the noise by relying on the difference between the target point cloud density and the noise point cloud density^11,12^. The radius filter is the most commonly used denoising method. It takes a point to be determined as the center, controls the size of a space by setting the radius, and analyzes the average distance from each point in the space to the center point of the space to measure the density of the space. The threshold determines whether the point is noise. This method can determine noise only through a fixed global threshold and is not suitable for changes in noise density^13,14^.

On this basis, the statistical filter adapts to discriminate noise of different density changes by determining the average distance information of the global neighborhood and obtaining the standard deviation distribution. Statistical outlier removal is a typical statistical filter^13^. However, the neighborhood space is still controlled by a fixed radius^15^. During evaluation of the noise point cloud, part of the point cloud in a target with a complex shape may be judged as noise, and the effective point may be removed by mistake^2^. Density-based spatial clustering of applications with noise (DBSCAN) is an algorithm that can cluster target point clouds of any shape in noisy point cloud scenes and can be well applied to point cloud clustering tasks of complex-shaped devices^16–18^. However, the computational efficiency of DBSCAN is not high, and DBSCAN is not suitable for substation scenarios where the amount of point cloud data is as large as several hundred megabytes^19–21^.

Algorithms for target recognition based on neural networks have developed rapidly and can be used to identify two- or three-dimensional targets, but they depend heavily on the establishment of training samples^22–24^. Many types and models of power equipment can be found in substations. Establishing enough training samples for each kind of equipment is a very large project that is difficult to accomplish in a short time. Particularly for power equipment of the same category but different types, their differences are often difficult to intuitively discover. Generating training samples and establishing the corresponding neural networks for subtle differences are great challenges that require the cooperation of professionals with both electric power and artificial intelligence knowledge.

The very large workload of generating training samples directly limits the mature application of 3D object recognition algorithms in substation equipment recognition^25–27^. The common two-dimensional image recognition algorithm can identify multiple targets at the same time and has been used to identify equipment in substations. A single-shot multibox detector (SSD) is a kind of target detection method with both speed and accuracy that is widely used in various fields of image recognition^28^. However, it has the disadvantage of not being robust to small targets, as the shallow feature map is not strong enough^29^. The basic network is changed from VGG to ResNet to improve the deeper network representation ability^30^. This direction is important to improve the SSD prediction capabilities. DenseNet, as a better CNN model of ResNet, offers greater improvements in SSD prediction^31–33^.

In this article, we proposed a cross-constrained hierarchical clustering algorithm based on traditional voxel clustering that reduces the dimensionality of 3D point cloud information to 2D, which not only improves the computational efficiency but also effectively removes noise. This algorithm uses the constraint criteria to accurately determine the noise and can process point cloud scenarios of large substation sites with hundreds of megabytes of data at a time. In this cross-constrained hierarchical clustering algorithm based on traditional voxel clustering, the 3D point cloud data is reduced to N 2D point cloud projection data, and each 2D point cloud data is clustered. If point cloud clusters exist in all slices at the same horizontal position, these clusters are retained. Otherwise, clusters at that horizontal position are considered noise and removed. Then, we applied the improved SSD structure to the image recognition of power equipment and quickly identified the main equipment category in a cluster. Finally, for equipment of the same category but of different types, we use the iterative closest point (ICP) algorithm for fine registration, finely distinguish the equipment type through match point distances, obtain the world coordinates of the model in the scene, and put the model into the scene^34,35^.

**Figure 1:**
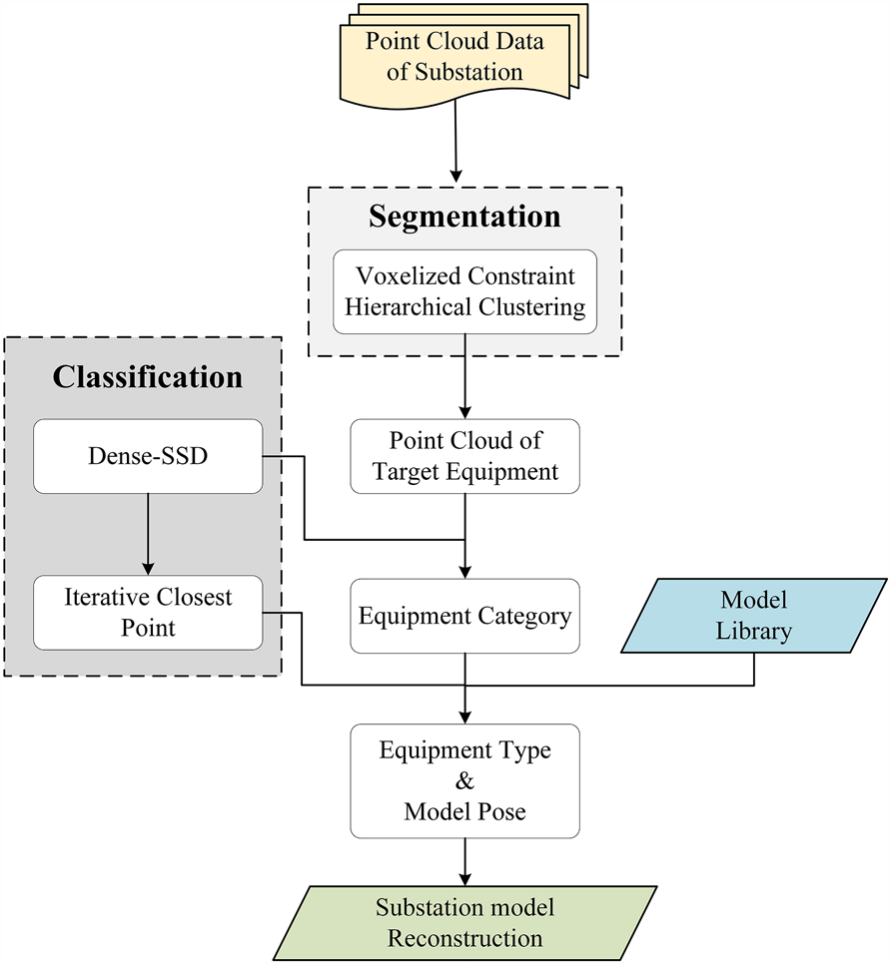
Workflow of substation point cloud scene automatic modeling.

## Methods

Our automatic modeling approach is shown in Fig. 1. First, we use voxelized constraint hierarchical clustering (VCHC) to segment the cloud scene of the substation site to obtain clusters containing one or more pieces of equipment. Then, we use Dense-SSD to identify the snapshot images of each point cloud cluster and obtain the category of equipment included in the clusters and the number of equipment. Finally, we retrieve the corresponding different types of models from the model library according to the equipment category, use the ICP to register each type of model with the target equipment point cloud, and determine the equipment type by the distance of the matched points. Furthermore, we can obtain the pose of the equipment model in the substation scene from the calculation results of the ICP to put the model into the actual substation scene.

### Voxelized constraint hierarchical clustering

**Figure 2:**
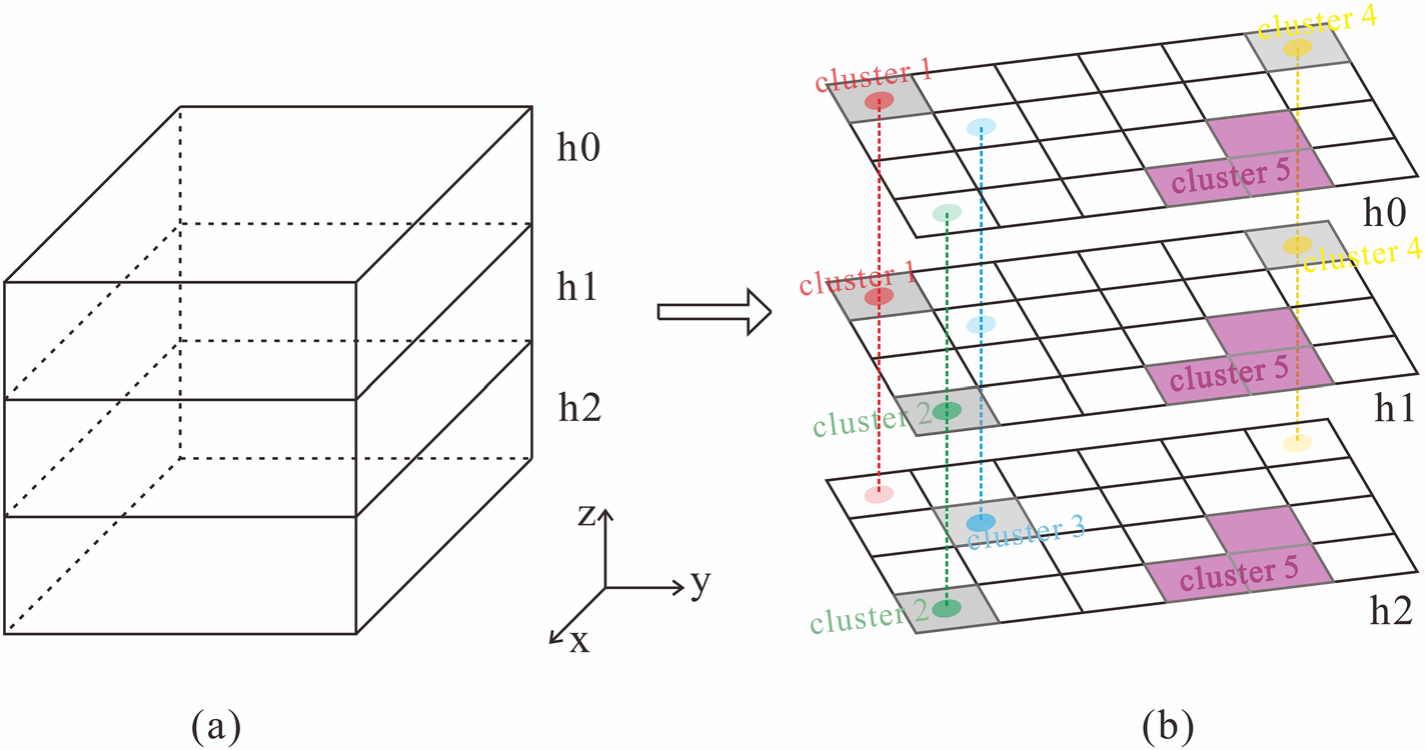
Schematic diagram of the longitudinal constraint: (**a**) shows that the whole area can be divided into three elevation intervals: h0, h1 and h2; (**b**) indicates that the point cloud data in different elevation intervals are projected to three planes. The gray areas represent the noise point cloud clusters, the purple areas represent the non-noise point cloud clusters, and the vertical point line connects the unified horizontal position unit.

According to the characteristics of the point cloud noise and vertical continuity of the equipment point cloud, we use the clustering results of different plane levels to constrain each one to identify and remove the noise point cloud. Point clouds of the same elevation interval are projected to the same plane, and the voxelization clustering algorithm is used for clustering. The clustering results of plane point clouds in different elevation intervals can be obtained, as shown in Fig. 2. The distribution of plane clusters corresponding to different height intervals is not completely consistent. In Fig. 2, cluster 1 appears only in the h0 and h1 cluster distribution planes and not in the h2 cluster distribution plane. According to the structure of the power equipment and the vertical discontinuity of the point cloud noise, cluster 1 can be judged as a noise point cloud and removed. By analogy, clusters 2, 3 and 4 at each plane can be judged as noise point clouds to be removed due to the inconsistent distribution of clusters in the cluster plane corresponding to different elevation intervals. For the area with the same cluster distribution (cluster 5), combined with the structure of the electrical equipment and the vertical continuity of the equipment point cloud, the point cloud in the area can be judged as an effective point cloud.

**Figure 3:**
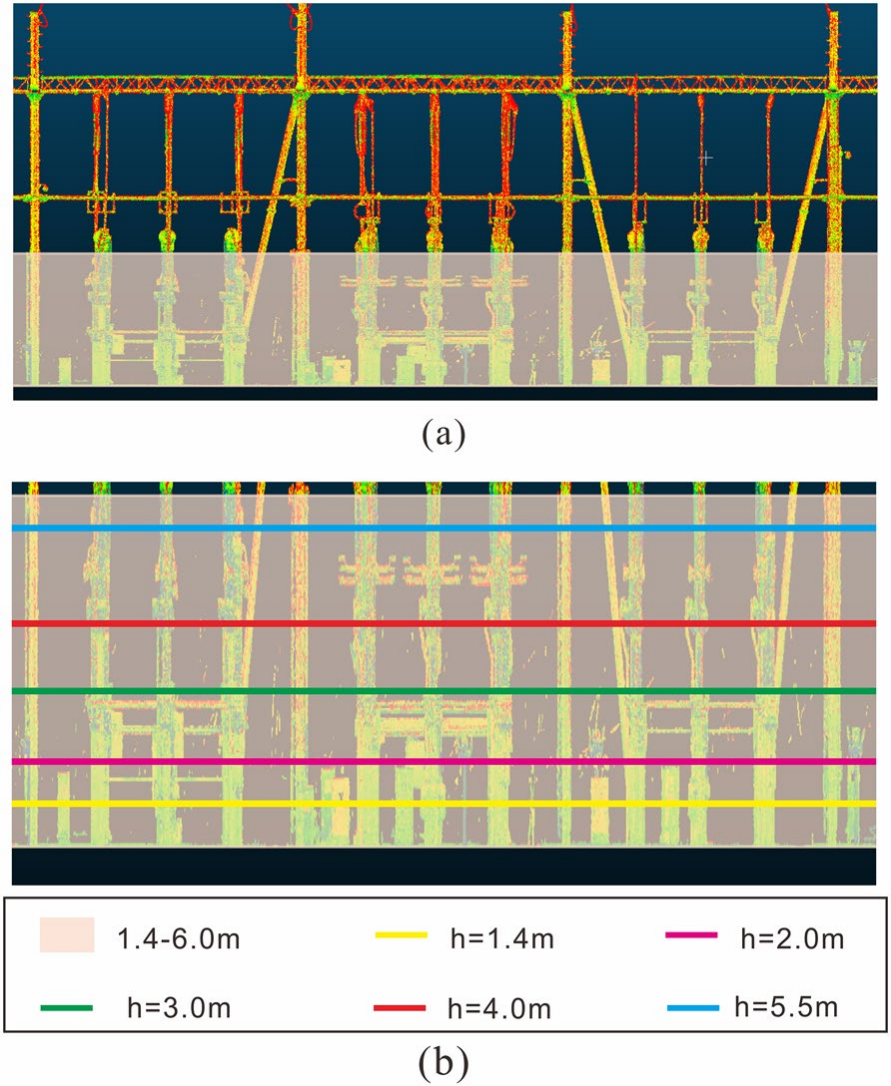
Key point cloud data interception and elevation interval division in a 220 kV area. (**a**) The key point cloud data interception; (**b**) the key point cloud data elevation interval division.

In practical applications, the size of each device in the substation are constructed in accordance with standard specifications, and the sizes of components such as bases, connecting rods, and insulator strings are also fixed. Therefore, we can fix the height intervals. We can obtain only the point cloud data with an elevation below 6 m (the elevation of the ground is 0 m) as the key point cloud data, as shown in Fig. 3a. This part of the point cloud contains the main part of the equipment. The key point cloud data can be further divided into intervals according to height interval considered (Fig. 3b). In the 220 kV area, the elevation of the connecting rod of the three-phase equipment is within the range of 2–3 m, which indicates the feasibility of reducing the dimension of the equipment shape characteristics to a two-dimensional plane.

After the noise point cloud clusters are removed by using the cross constraint, the remaining point cloud clusters need to be merged. We use the recombination algorithm to recluster the bounding boxes with a hierarchical strategy:

**Table:** 

**Algorithem 1:** Recombination the voxel grids	
**Input**: boxes $B=\{B_i | i = 1, 2, 3, \cdots , N\}, B_i=\{x_{i\_min}, x_{i\_max}, y_{i\_min}, y_{i\_max}\}$	
**Output**: boxes $B^{\prime }=\{B^{\prime }_j | j = 1, 2, 3, \cdots , M\}, B^{\prime }_j=\{x_{j\_min}, x_{j\_max}, y_{j\_min}, y_{j\_max}\}$	
1. Preparing the target box $B^{\prime }$, adding *B*1 into $B^{\prime }$1 and removing *B*1 from *B*.	
2. Beginning a loop for *B*. Setting $j=0$ at the beginning. Setting the length of *B* to 0 as the terminal condition.	
2.1 Selecting the last element of $B^{\prime }$ as a comparative box b. Setting a set Btemp and putting *b* into $B_{temp}$ as the $1^{st}$ element.	
2.2 Beginning a loop for *B*. Setting $i=0$ for the beginning. The terminal condition is when the loop is at the end of *B*.	
2.2.1 Determining whether $B_i$ intersects *b*. If an intersection exists, then add $B_i$ to Btemp and remove $B_i$ from *B*.	
2.3 Determining whether the length of Btemp has increased. If the length has increased, then calculate the maximum outer bounding box (merging the newly added boxes into one box) and add it to *B*.	
2.4 If the length has not increased, add $B_1$ to $B^{\prime }_1$ and remove $B_1$ from *B*.	

This hierarchical clustering strategy is an extension of the voxel clustering strategy and can reduce the number of clusters. The shape of the box conforms to the distribution law of electrical equipment in the substation scene.

### Dense-SSD

We added a network for extracting deep features on the basis of SSD and built a new neural network, similar to what the Dense-SSD (DSOD) does^28,33,36^, that predicts the type of equipment based on point cloud images of the power equipment. In this detection framework, we extract deep features through DenseNet and then combine the target frame suggestion strategy and frame regression algorithm in the SSD algorithm to rebuild an end-to-end object recognition and detection network^36^. The Dense-SSD algorithm has a high accuracy rate for detecting small objects, can identify the divided power equipment and the parts around the equipment that are not part of the equipment, including pedestrians and other equipment, and reduce the adverse effects of oversegmentation on equipment recognition. The Dense-SSD model is divided into two parts: a feature extraction part and a prediction output part.

The feature extraction part has a DenseNet structure, including a stem block, four dense blocks, two transition layers, and two transition w/o pooling layers.

In the stem block, to reduce the information loss of the original input image, we replaced the large convolution kernel with several small convolution kernels and changed the original design (7 $\times$ 7 convolution layer, stripe = 2, 3 $\times$ 3 maximum pooling layer, stripe = 2) in DenseNet to a combination of four 3 $\times$ 3 convolution layers and 2 $\times$ 2 maximum pooling layers.

The transition layer between two adjacent dense blocks is composed of a 1 $\times$ 1 volume layer and a 2 $\times$ 2 maximum pooling layer, which realizes the compression model and reduces the number of characteristic graphs output by each dense block. The transition w/o pooling layer ensures that the number of dense blocks is increased without reducing the resolution of the final feature map and is composed of 1 $\times$ 1 convolution layers.

The prediction output layer retains the structure of the SSD network prediction layer, predicts the prediction confidence of all object categories and the position offset value of the prediction frame, and combines the side output feature map of the stem block and dense block as shallow information and the final output feature map of the network as deep information. The shallow feature and deep feature jointly determine the predicted value and realize deep supervision.

**Figure 4:**
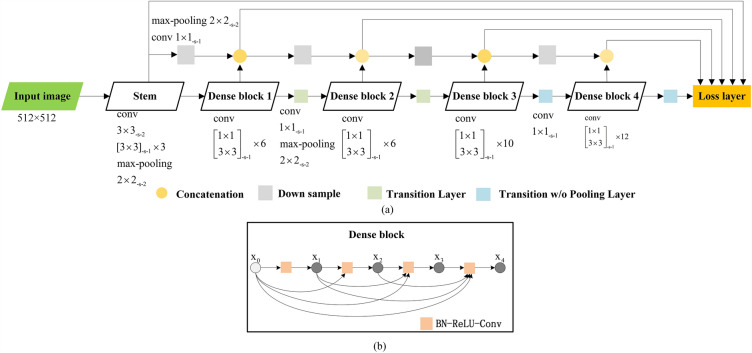
Key point cloud data interception and elevation interval division. (**a**) Shows the overall structure of the Dense-SSD network, (**b**) a schematic diagram of the dense block structure.

To reduce the amount of calculation, each feature map is downsampled and merged in turn. The downsampling module includes the maximum pooling layer and the convolution layer. The overall structure of the Dense-SSD network and a schematic diagram of the dense block structure are shown in Fig. 4.

In the whole structure (Fig. 4a), the dense block is the core part. The connection mode introduces a direct connection from any layer to all subsequent layers. The output of the upper layer is x0, x1, x2, x3, and x4. The connection structure between outputs is composed of a batch normalization (BN) layer, corrected linear unit (ReLU) and 3 $\times$ 3 containment (conv).

The dense blocks (Fig. 4b) realize the reuse of shallow feature maps by establishing short-circuit connections between all the front layers and the back layers. In traditional deep convolutional neural networks, as the number of network layers increases, the gradient signals may gradually become very small, making it challenging for the model to converge during the training process. This phenomenon occurs because in deep networks, information needs to pass through multiple non-linear layers, which can lead to an exponential decrease in gradients, ultimately causing the problem of vanishing gradients. Short-circuit structures, on the other hand, allow the network to maintain some original information of low-level features through identity mappings during the learning process. Consequently, if the features learned in subsequent layers do not provide more useful information compared to the previous layers, the network can opt to skip certain layers and directly propagate the low-level features.

## Results

### Clustering results of VCHC

We compare our clustering method with DBSCAN and voxelization clustering in terms of the segmentation time and accuracy on point cloud data at different voltage levels. The segmentation accuracy is the ratio of the number of correctly segmented devices to the total number of segmented devices. The comparison results are shown in Table 1.

**Table 1:** Comparison of efficiency and accuracy of different Segmentation methods for substation point cloud scenes in different voltage levels.

	Times (s)			Accuracy (%)		
	35 kV	220 kV	500 kV	35 kV	220 kV	500 kV
DBSCAN	123.84	157.88	884.39	16.58	27.40	36.51
Voxelization clustering	1092.45	1347.26	947.88	44.31	38.44	42.18
VCHC	12.81	15.12	20.89	83.00	96.12	91.54

According to the calculation time results in Table 1, voxelization clustering is the most inefficient scene segmentation method. The calculation efficiency of DBSCAN is higher than that of voxelization clustering, but with the increase in voltage level, the point cloud scene becomes increasingly larger, the number of point clouds increases, and the advantage of DBSCAN becomes increasingly less obvious.

The calculation efficiency of VCHC is significantly higher than the calculation efficiency of the other two, and the calculation time does not change significantly with the voltage level, which shows that the method is less affected by the number of point clouds. The efficient segmentation calculation efficiency of VCHC might come from its special dimensionality reduction processing.

**Figure 5:**
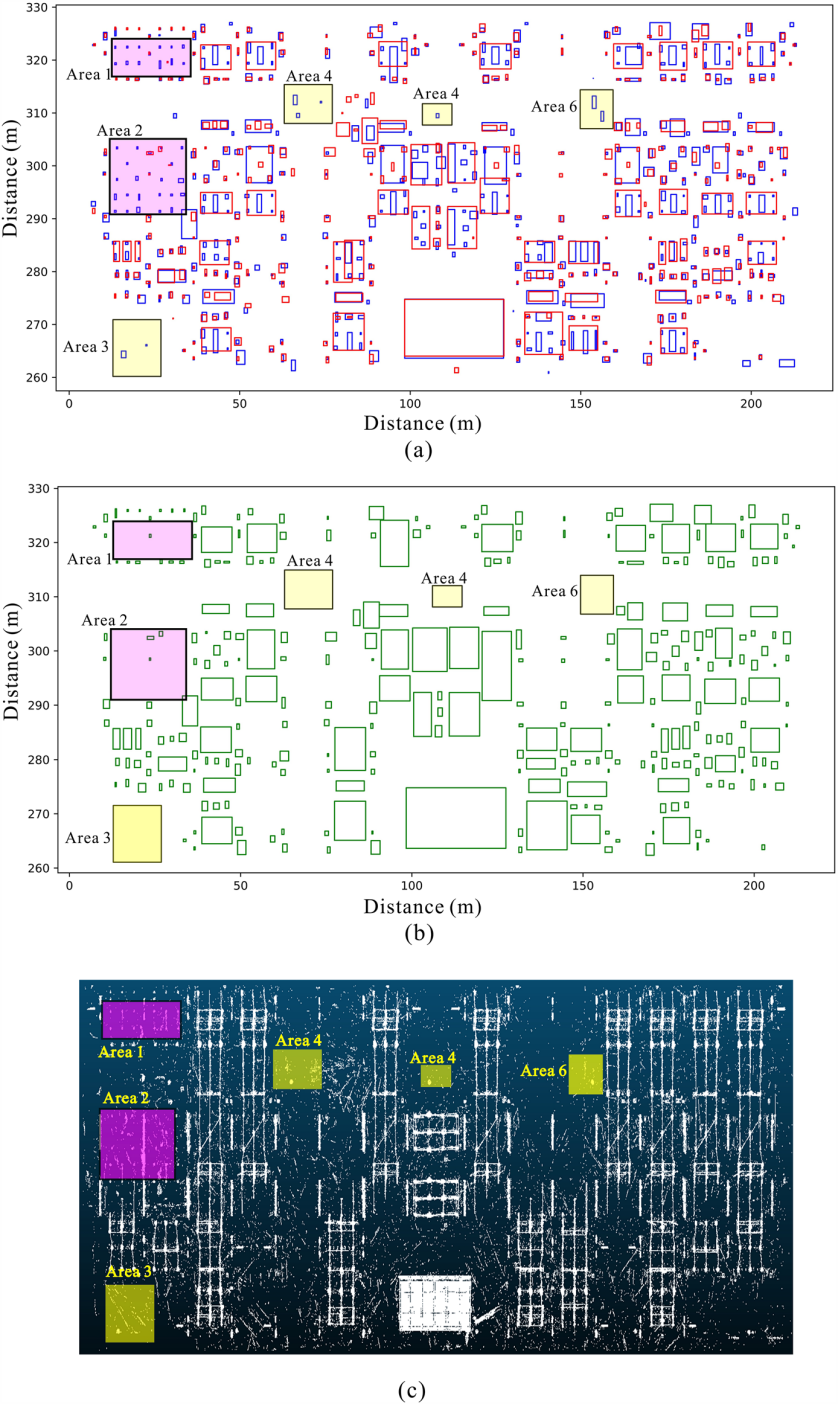
Constraint process and clustering results of the measured point cloud scene in the 220 kV area. (**a**) The bounding boxes of clusters in two elevation intervals. The blue box represents the bounding box in the 1–2 m interval, and the red box represents the bounding box in the 2–3 m interval. (**b**) The new bounding boxes generated by merging the clusters of two intervals after applying the cross constraint. (**c**) The top view of the measured point cloud data. The areas in red contain a large number of point clouds of equipment under construction. The areas in yellow contain a large number of noise point clouds.

In the cloud scene of substation sites, the sparse and scattered noise point cloud in substations can be easily identified and removed by the traditional denoising method. However, two kinds of noise are difficult to identify: superdensity observation noise and construction equipment point clouds. When these two kinds of noise are not recognized, the clustering segmentation algorithm easily clusters them into point cloud clusters, which will frustrate device recognition. We use the point cloud clustering of a 220 kV substation to illustrate the ability of our denoising method to suppress this noise (Fig. 5).

We selected the point cloud data in two elevation intervals, a 1–2 m interval and a 2–3 m interval, for voxelized clustering on the plane. The two-dimensional clustering results are shown in Fig. 5a. The outer bounding boxes of these two elevation intervals intersect in multiple places in the horizontal direction. The result of the cross constraint calculation is shown in Fig. 5b. However, disjoint places also exist, and six example areas in these places are marked in Fig. 5. The areas in red contain a large number of point clouds of equipment under construction. The areas in yellow contain a large number of noise point clouds.

From the top view of the measured point cloud, obvious noise point clouds can be seen in marked areas 3–6. These noise point clouds are dense, have a large extension range and are difficult to distinguish from the device point cloud by density and extension range.

In marked areas 1 and 2, much of the equipment construction has not been completed, and only concrete pillars support the equipment at the substation site. This observation also explains why more clusters exist in the 1–2 m interval than in the 2–3 m interval.

### Segmentation results comparison

The comparative method is DBSCAN, as this density-based clustering approach can effectively remove noise and avoid unfairness that might arise from other clustering methods requiring prior noise removal. We designed multiple DBSCAN classifiers with neighborhood radii $\varepsilon$ set to 0.1 m, 0.5 m, and 1.0 m, along with minimum point counts *MinPts* set to 30 and 50, respectively, to perform the clustering segmentation task. We then recorded their segmentation accuracy and compared it with the segmentation accuracy of the proposed VCHC method (Table 2)

**Table 2:** Segmentation accuracy comparison of different methods for substation point cloud scenes.

Voltage level	Number of devices to be built	Data size (GB)	Method		Run time (min)	Number of segments	Number of devices	Acc (%)
35kV	184	15.36	VCHC		26	168	153	83.152
			$\varepsilon$ = 0.1	*MinPts* = 30	325	344	–	–
			$\varepsilon$ = 0.5		532	279	–	–
			$\varepsilon$ = 1.0		768	152	31	16.85
			$\varepsilon$ = 0.1	*MinPts* = 50	361	339	–	–
			$\varepsilon$ = 0.5		573	261	–	–
			$\varepsilon$ = 1.0		802	148	29	15.760
220kV	206	14.80	VCHC		30	203	198	96.116
			$\varepsilon$ = 0.1	*MinPts* = 30	367	331	–	–
			$\varepsilon$ = 0.5		612	265	–	–
			$\varepsilon$ = 1.0		906	166	56	27.368
			$\varepsilon$ = 0.1	*MinPts* = 50	440	319	–	–
			$\varepsilon$ = 0.5		658	224	–	–
			$\varepsilon$ = 1.0		951	159	5	27.368

The segmentation clusters obtained by the proposed VCHC method were the closest in number to the actual number of devices to be modeled. The next closest was the number of segmentation clusters obtained by DBSCAN with a neighborhood radius $\varepsilon$ of 1.0 m. However, when the neighborhood radii $\varepsilon$ were set to 0.1 m and 0.5 m, the number of segmentation clusters obtained was significantly higher than the actual number of devices. This was due to a large amount of noise present in the clustering results, causing DBSCAN to incorrectly cluster noise as valid device point clouds.

Even with the proposed VCHC method, which achieved the closest number of segmentation clusters to the actual devices, there were still some clusters formed by noise point clouds and some over-segmented clusters. Ultimately, VCHC achieved a segmentation accuracy of 83% for the 35kV substation point cloud scenes. This accuracy improved to 96% for the 220kV substation point cloud scenes. This improvement was due to the generally larger equipment size in the 220kV substations compared to the 35kV substations, making noise easier to be effectively recognized.

On the other hand, for DBSCAN, although the number of segmentation clusters obtained with a neighborhood radius $\varepsilon$ of 1.0 m was relatively close to the number of devices to be modeled, the number of well-formed device point cloud clusters was still quite low. In the 35kV substation scenes, they only accounted for about 15%, and in the 220kV substation scenes, they only accounted for about 27%.

### Comparative analysis of VCHC

**Figure 6:**
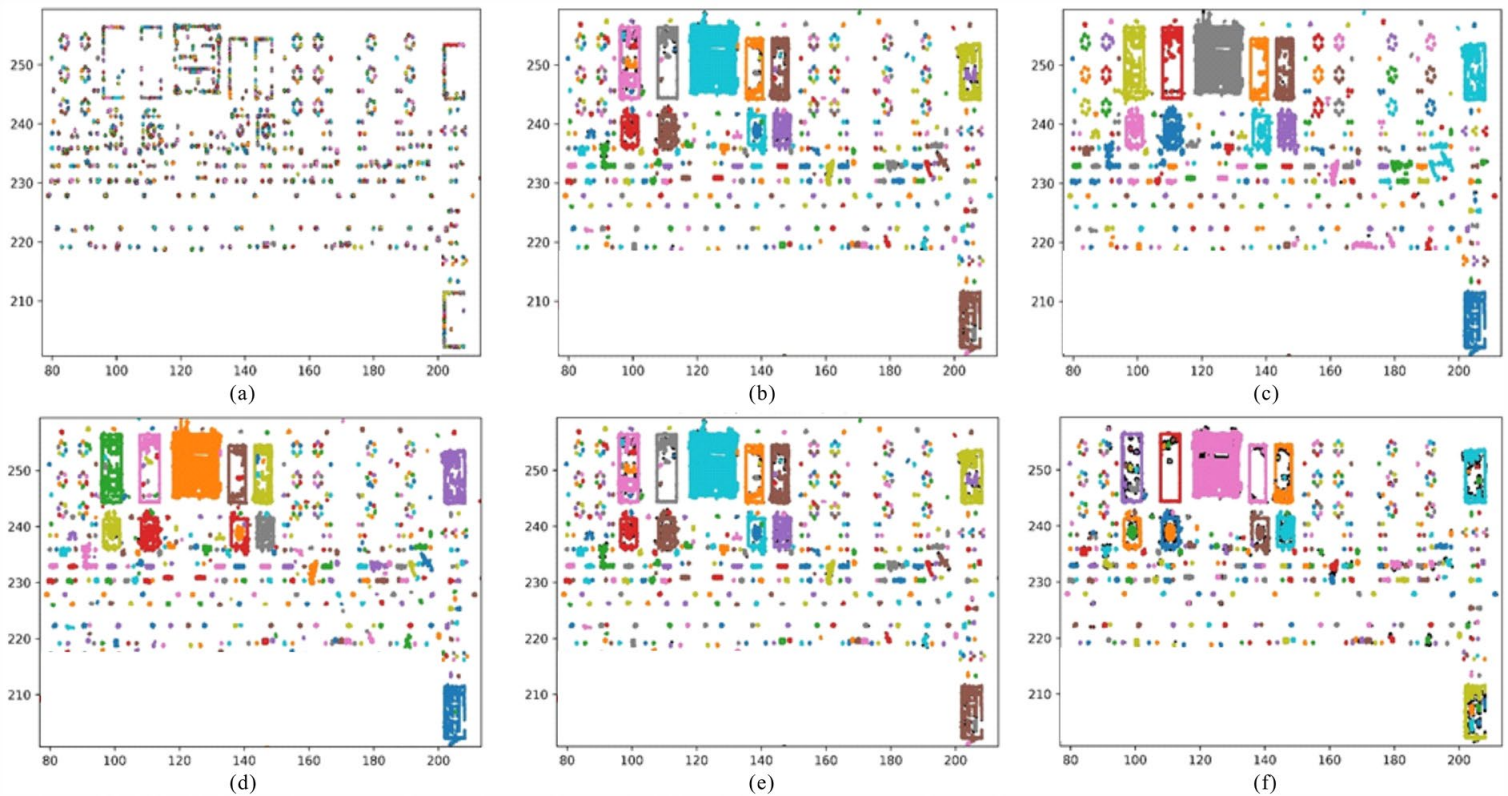
The clustering results obtained from applying DBSCAN to the 35kV area with different values for neighborhood radius $\varepsilon$ and minimum neighborhood point count *MinPts*. (**a**)–(**c**) respectively display the DBSCAN clustering results with a minimum neighborhood point count of 30 and $\varepsilon$ of 0.1 m, 0.5 m, and 1.0 m. (**d**)–(**f**) respectively display the DBSCAN clustering results with $\varepsilon$ = 0.5 m and minimum neighborhood point counts of 10, 50, and 80.

In DBSCAN, when setting the neighborhood radius $\varepsilon$, it must exceed the minimum distance between points for points to exist within the neighborhood. To ensure effective noise reduction, the neighborhood radius should not be too large either, as an excessively large radius may retain too much noise, hindering the clustering process. We conducted two sets of comparisons: one compares the clustering results for different neighborhood radii $\varepsilon$, and the other compares the clustering results for different minimum neighborhood point counts (*MinPts*). The clustering result comparisons are shown in the Figure 6.

In the comparison of clustering results for different neighborhood radii, with neighborhood radii values of 0.1 m, 0.5 m, and 1.0 m, and a minimum neighborhood point count of 30, we observed the following patterns:

1. Smaller neighborhood radii lead to more points being classified as noise, resulting in a larger number of clusters. This causes the point cloud of the equipment to be segmented into multiple smaller clusters. For $\varepsilon$ = 0.1, most equipment points, apart from noise, are treated as noise and removed. The remaining equipment points are divided into very fine-grained clusters, leading to the fragmentation of points belonging to the same equipment.
2. As the neighborhood radius increases, the number of clusters and noise points decrease, and there are fewer instances of equipment points being incorrectly segmented. However, if the neighborhood radius becomes too large, most noise points are included in equipment clusters and retained. Additionally, points belonging to different equipment may be assigned to the same cluster, leading to poor clustering results. This behavior is primarily due to the fact that when the neighborhood range is small, a significant number of non-noise points are subdivided excessively. Since the minimum point count is fixed, clustering results with smaller neighborhoods mistakenly identify many excessively subdivided equipment points as low-density noise. On the other hand, setting a larger neighborhood range disregards the distance between different equipment points, causing points that are close but do not belong to the same equipment to be grouped together, along with some low-density noise points being retained.

**Figure 7:**
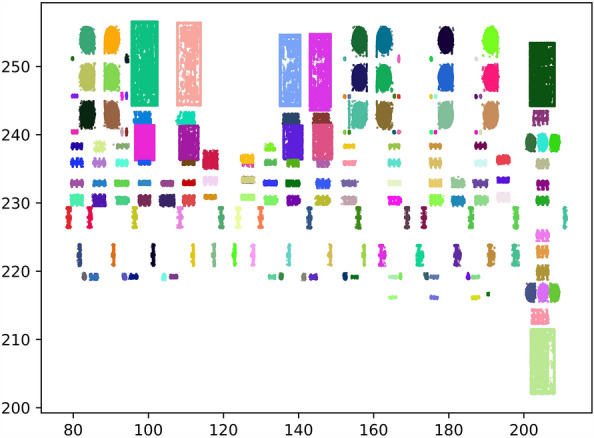
Clustering results for VCHC segmentation of 35 kV area.

In the comparison of clustering results for different minimum neighborhood point counts (*MinPts*), with a neighborhood radius ($\varepsilon$) of 0.5 m, and *MinPts* values of 10, 50, and 80, we observed the following: from the clustering results, for the same neighborhood radius, different *MinPts* values lead to a relatively consistent number of clusters and overall partitioning. However, the choice of *MinPts* determines whether the clusters represent equipment points or noise. It can be seen that the partitioning results for different *MinPts* values show minor differences (Fig. 6). Nevertheless, as *MinPts* increases, more equipment points are mistakenly deleted, while setting *MinPts* too low leads to an increase in retained noise points.

The segmentation results obtained through the VCHC method exhibit minimal residual noise, and the clustering outcome is clear and clean (Fig. 7).

### Classification results

We use the Dense-SSD to classify the power equipment categories and ICP registration to classify the type of equipment. The classification results are shown in Table 3. The confidence value in the category classification is the softmax loss function of the classification confidence in the SSD. The classification accuracy is the ratio of the number of equipment correctly classified to the total number of equipment to be classified. In the substation point cloud scenario, the number of devices to be modeled and the number of correctly modeled devices are shown in Table 3. The total number of correctly modeled equipment is divided by the total number of equipment to be modeled, resulting in a modeling achievement rate of 92.86%

**Table 3:** Statistics on evaluation of modeling confidence and accuracy. Total value is ${\frac{1}{N}\sum _{n=1}^{N}(\sum _{m=1}^{M}v)}$ , *M* is the total number of types for a category, and *N* is the total number of equipment types.

Equipment	Number of equipment to be modeled	Confidence in category classification	Number of correctly modeled equipment	Accuracy in type classification (%)
Current transformer	103	0.97	99	96.117
Potential transformer	102	0.97	98	95.078
Capacitor voltage transformer	60	0.97	58	96.667
Insulator pillar	101	0.98	95	94.059
Circuit breaker	61	0.95	57	93.443
Isolation switch	60	0.88	52	86.667
GIS	12	0.84	12	100.00
Lightning arrester	80	0.97	77	96.250
Capacitor	11	0.85	5	45.455
Reactor	13	0.86	7	53.846
Total	603	0.95	560	92.869

In the actual modeling process, it is often difficult for us to distinguish current transformers, voltage transformers and capacitor voltage transformers based on point cloud images alone. Distinguishing these transformers requires the help of on-site photos and CAD construction drawings. Therefore, we put these transformers into the same category, with further differentiation occurring in the matching process.

**Figure 8:**
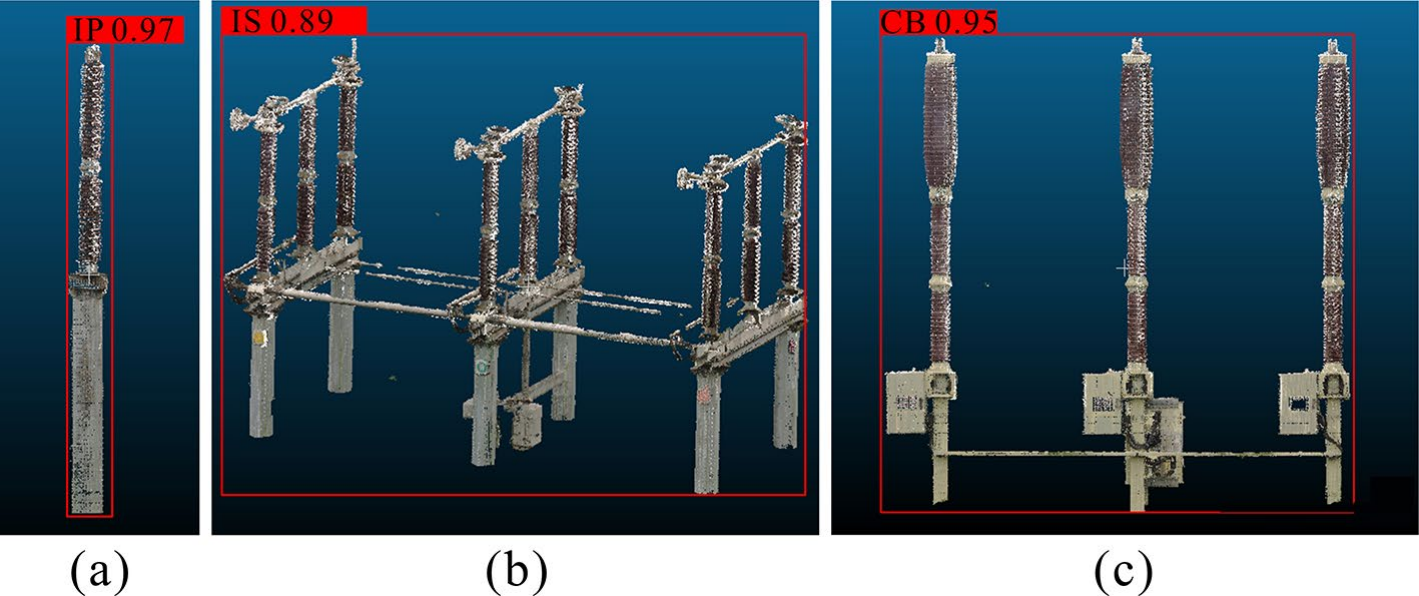
Equipment category classification results. (**a**) The identification result of the insulated pillar (IP), (**b**) the identification result of the isolating switch (IS), and (**c**) the identification result of the circuit breaker (CB). The red frames are the candidate boxes generated after identifying the equipment, and the value at the top of the candidate box is the confidence.

The category classification results of the three main pieces of equipment are shown in Fig. 8. The candidate frame accurately outlines the spatial position of the equipment and gives a large value of confidence. We can use the confidence value to directly distinguish the category of equipment.

In the entire substation scene, six categories of insulation pillars, eight categories of circuit breakers, and 26 categories of isolation switches can be observed. The greater the number of categories, the more difficult the NN training process is, and the lower the confidence value of the prediction will be. Therefore, the confidence of the insulator pillar is the highest, and the confidence of the isolating switch is the lowest.

While increasing the number of device models of the same category in the training set can make the training of neural networks more challenging and result in lower confidence, the confidence level still remains significantly higher compared to the confidence with devices of other categories. Therefore, we can accurately determine its category based on the highest confidence level. Even though the confidence level of the isolation switch is less than 90%, we can still confidently identify its category based on the available confidence level.

**Figure 9:**
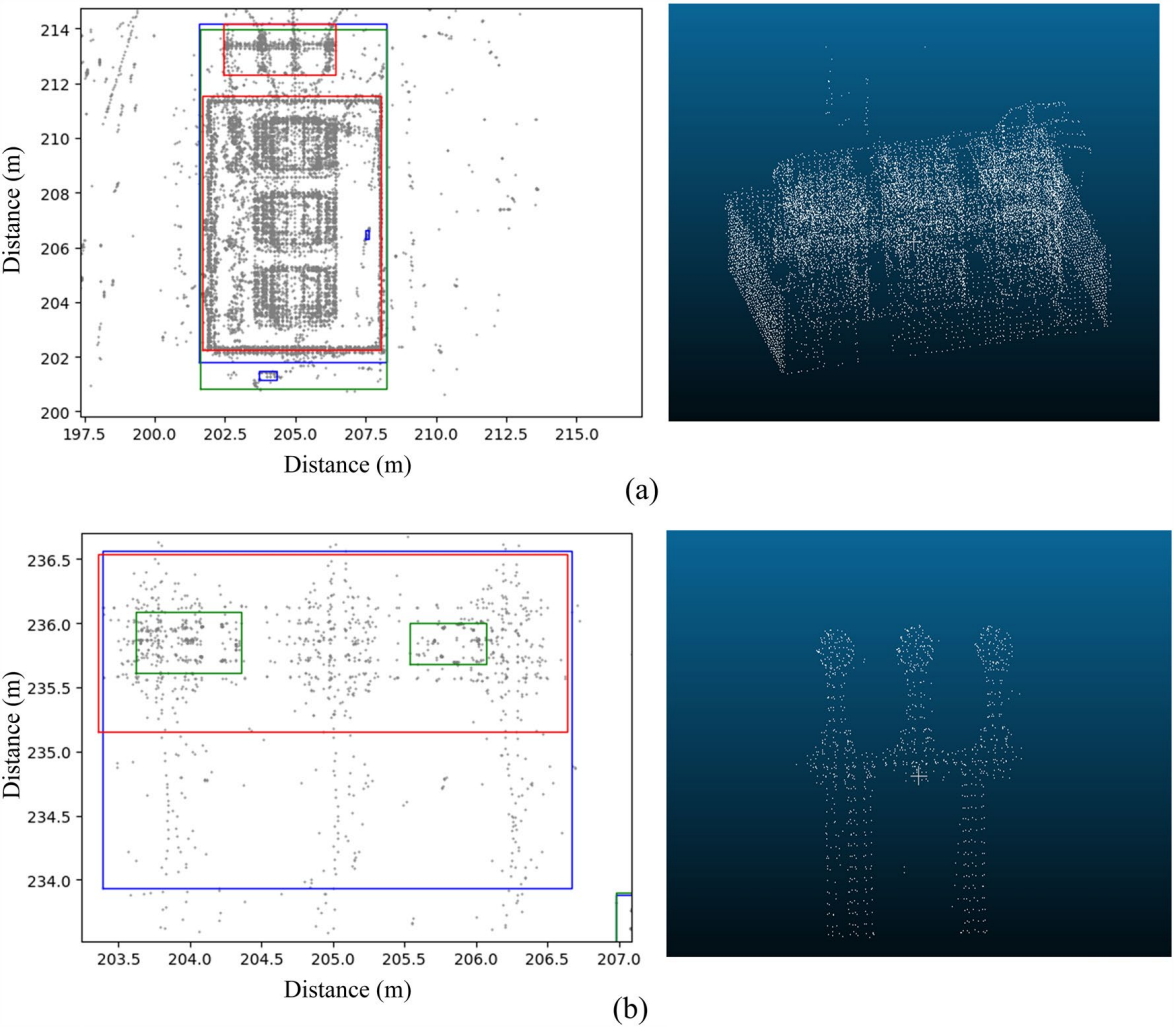
Clustering results of equipment point clouds. (**a**) The clustering result and point cloud data of the capacitor bank; (**b**) the clustering result and point cloud data of the isolation switch. The green frames represent the bounding boxes of the DBSCAN clustering results, blue frames represent the bounding boxes of the voxelized clustering results, and red frames represent the bounding boxes of the clustering results generated by our clustering method, VCHC.

### Recognition results comparison

We used the point cloud clusters from the VCHC segmentation results as the basis for recognition. We compared the recognition results of our proposed Category Recognition & Type Fine Matching approach with the results from traditional VGG, AlexNet, ResNet, and DenseNet models (Table 4). During the Category Recognition stage, there was little variation in the recognition results across all methods, staying at approximately 96–97%. This indicates that traditional CNN-based neural networks can effectively identify different equipment categories based on their appearances.

When it comes to cases where the category is the same but the type is different, meaning the overall appearance is similar but with different details, the fine matching approach significantly widened the gap between our proposed method and other traditional methods in terms of recognition accuracy. Our proposed method achieved a final recognition accuracy of over 95%, and for the 220 kV substation scenes, the recognition accuracy reached 97%. In contrast, the recognition accuracy of other neural networks was around 30%.

**Table 4:** Recognition cluster recognition accuracy comparison of different methods for substation point cloud scenes.

Voltage level	Number of devices to be identified	Method	Identify category of devices		Identify type of devices	
			Number	Acc (%)	Number	Acc (%)
35kV	168	Our method	163	97.024	160	95.238
		VGG	159	94.643	44	26.190
		AlexNet	160	95.238	44	26.190
		ResNet	162	96.429	47	27.976
		DenseNet	163	97.024	47	27.976
220kV	203	Our method	198	97.537	197	97.044
		VGG	196	96.551	72	35.468
		AlexNet	196	96.551	71	34.975
		ResNet	198	97.537	72	35.468
		DenseNet	198	97.537	71	34.975

## Discussion

### Using VCHC to prevent oversegmentation

Figure 9 shows the point cloud clustering results of capacitor banks and isolation switches in the scene from the top view. The green frames represent the bounding boxes of the DBSCAN clustering results, blue frames represent the bounding boxes of the voxelized clustering results, and red frames represent the bounding boxes of the clustering results generated by our clustering method.

For point clouds around the capacitor bank in Fig. 9a, the clustering results of VCHC show that two clusters exist: one is a capacitor bank, and the other is an isolation switch. The clustering results of DBSCAN and voxelized clustering are similar and cannot distinguish capacitor bank point clouds from adjacent isolation switch point clouds.

For point clouds around the isolation switch in Fig. 9b, the bounding boxes of the clustering results of voxelized clustering and VCHC completely contain the main part of the isolating switch, but the area delineated by the bounding box of VCHC is more accurate. The bounding box of voxelized clustering is affected by part of the noise point cloud. Although this part of the point cloud is sparse, the distance between each point is just less than the preset threshold distance of voxel clustering; hence, these noise point clouds and equipment point clouds are clustered into an integral cluster. In this case, the clustering results of VCHC are much more stable.

From Fig. 9b, compared with these two clustering methods based on distance information, the stability of DBSCAN clustering based on density information is worse. The clustering results of DBSCAN are concentrated in the middle area with a high density of point clouds, and oversegmentation appears.

The substation contains many pieces of three-phase equipment with connecting rods, and the point cloud density of these connecting rods is often much sparser than that of the main body of the equipment. For high-density noise, the point cloud density of these connecting rods is sparser than the density of the surrounding noise. The traditional method of judging noise based on the density difference easily misjudges the point cloud of the connecting part as noise, and the integrity of the point cloud of the three-phase equipment is broken in the denoising process. This situation easily leads to oversegmentation, which increases the computational difficulty of subsequent identification and matching.

For a point cloud scene with similar point cloud density, our method can stably judge the noise point cloud and the effective device point cloud. In our clustering and segmentation algorithm, the distance threshold we rely on is set according to the device interval, often to 0.2 m. This setting is chosen because by default, the minimum distance between devices in the substation is not less than 0.2 m, but in actual applications, the distance between the devices is usually above 2 m, and the threshold we set has high stability. For a point cloud scene with similar point cloud density, our method can stably judge the noise point clouds and the effective equipment point clouds.

**Figure 10:**
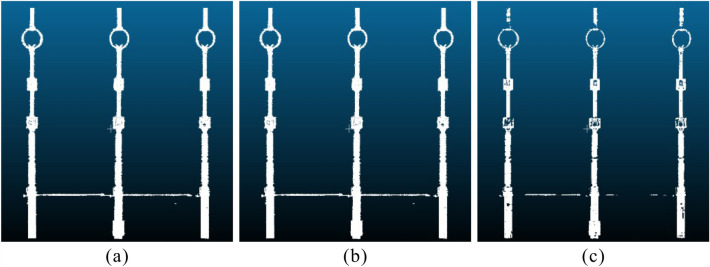
Segmentation results of isolation switch point clouds. Extracting point clouds of isolation by different methods. (**a**) and (**b**) present the segmentation results of VCHC, where the threshold distance used by VCHC in (**a**) is 0.2 m and the threshold distance used by VCHC in (**b**) is 1 m. (**c**) The denoising result of SOR, where the number of neighborhoods is 30 and the multiple of standard deviation is 1.

Assuming the threshold distance is 0.2 m and 1 m, respectively, compared with the two VCHC results calculated by different threshold distances (Fig. 10a and b), the segmented device point cloud is basically unchanged. This observation shows that the segmentation result is always stable when the selection of the threshold distance is not strict. By comparing the equipment point clouds with the point clouds (Fig. 10c) directly processed by Statistical Outlier Removal (SOR), we find that the equipment point clouds processed by SOR are incomplete, especially when the connecting rod part is obviously disconnected. We can infer that this degree of disconnection will result in oversegmentation.

### Classifying undersegmented clusters with Dense-SSD

**Figure 11:**
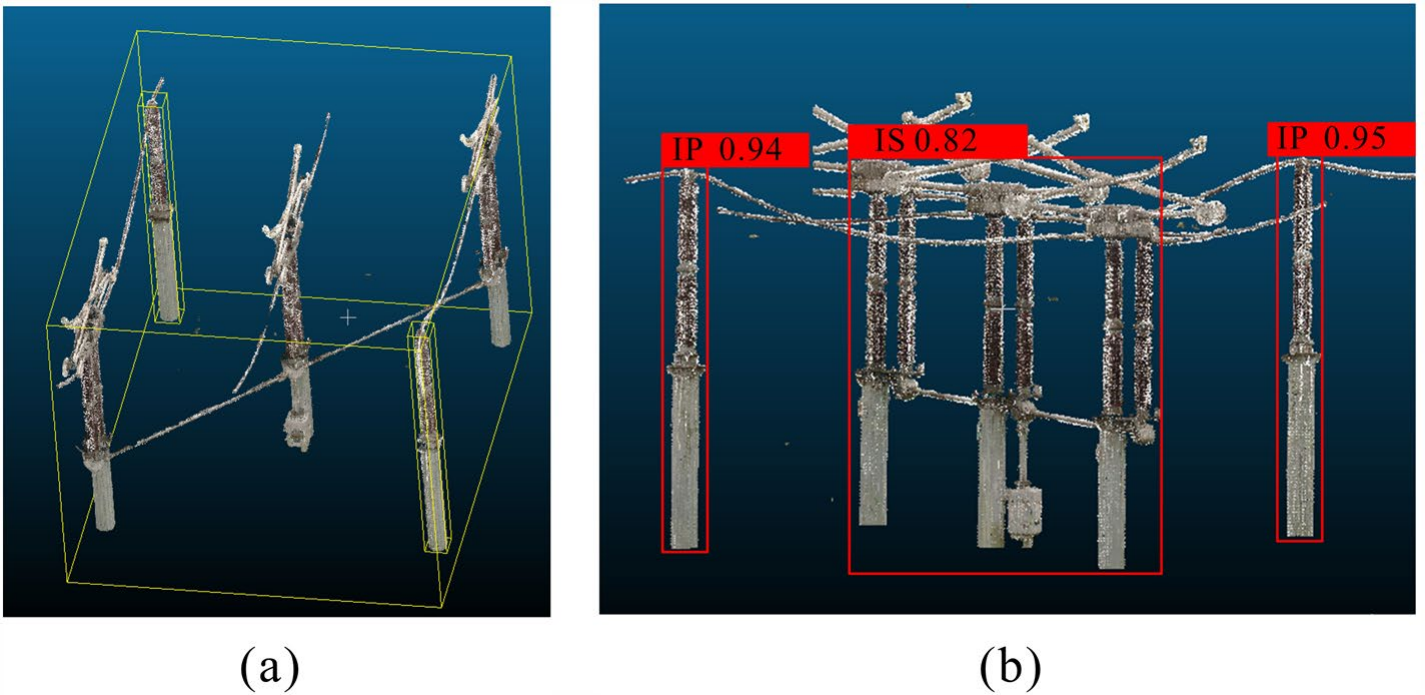
The result of identifying a cluster with multiple equipment. (**a**) The side view of the segmented cluster, where the yellow frames represent the bounding boxes of each piece of equipment. The bounding boxes of the insulating pillar intersect with the bounding box of the isolating switch. (**b**) The recognition result of the Dense-SSD. The red frames are the candidate boxes generated after identifying the equipment, and the value at the top of each candidate box is the confidence.

Although VCHC can avoid oversegmentation, it cannot avoid undersegmentation. In the bounding box cross constraint strategy, when the bounding boxes of multiple clusters contain intersecting parts, each cluster will be merged into one cluster. As shown in Fig. 11a, the bounding boxes of the insulating pillars and the isolating switch intersect with each other, and the final clustering result Fig. 11b is that a point cloud cluster contains two insulating pillar point clouds and one isolating switch point cloud.

The cluster contains multiple pieces of equipment, and our classification networks can accurately identify the insulation pillars and isolation switch in each cluster; the amount of each piece of equipment can also be correctly identified. This successful recognition can guarantee the effectiveness of subsequent matching calculations.

A cluster containing multiple devices is the result of undersegmentation. The Dense-SSD can effectively identify multiple devices in an undersegmented cluster, which shows that we can compensate for the shortcomings of VCHC in the identification step.

**Figure 12:**
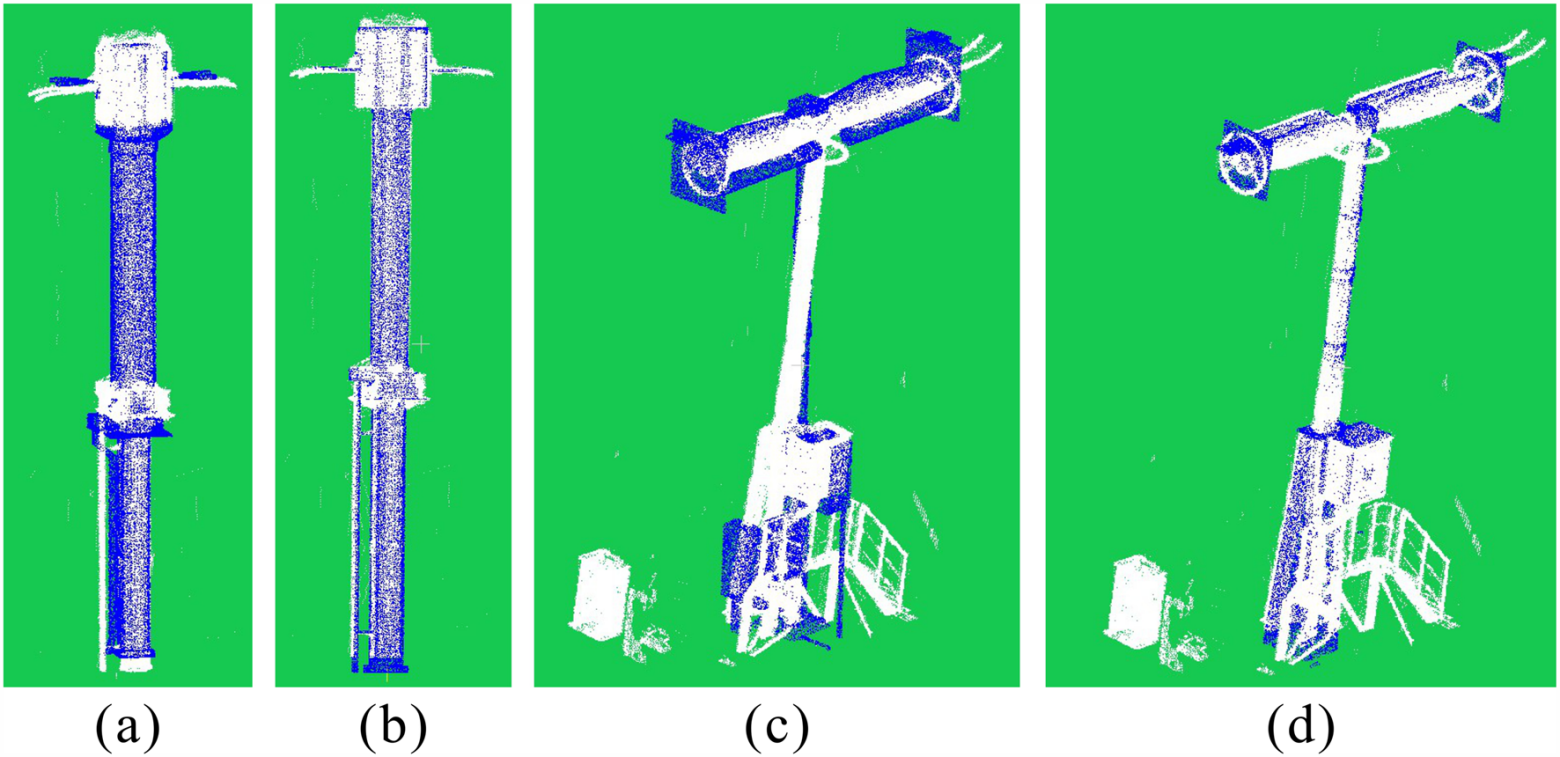
ICP registration results of different types of equipment. (**a**) and (**b**) The registration results of the current transformer; the RMSE of the matching distance is 0.1 and 0.05, respectively. (**c**) and (**d**) The results of circuit breaker registration; the RMSEs of the matching distance are 0.14 and 0.07, respectively. The blue point clouds are generated by the standard model in the model library, and the white point clouds are the measured data.

### Fine classification and model pose

Some equipment of the same category but different types have similar appearances. When the number of training samples is insufficient, training classification networks with high recognition efficiency in a short period of time becomes difficult. For example, the two types of current transformers shown in Fig. 12a and b are basically similar in appearance, with only slight differences in the middle part. The neural networks recognize that this subtle difference requires a heavy training process. However, the proximity of the two sets of point clouds to the ICP can be a good measure of the difference in the appearance of the two types of equipment.

When we use the Dense-SSD to identify these two types of current transformers, the confidence of the prediction is usually on the same level. However, the root mean square error (RMSE) of the matching distance given by the ICP registration is twice as different.

In the substation scene, some ancillary devices will exist around the main part of the power equipment. The usual modeling process does not have requirements for these ancillary devices. This ICP registration can avoid the interference caused by these accessory devices. In Fig. 12c and d, power boxes and stairs can be observed around the circuit breaker, but the final registration result can still accurately match the equipment type, with the smallest matching distance RMSE. The registration result is not interfered with by these accessory devices.

According to the registration result, we can not only identify the equipment type but also obtain the pose of the corresponding model in the substation scene. We can also effectively evaluate the modeling accuracy based on the RMSE distance in the registration calculation.

## Conclusions

We have proposed a model-driven method for reconstructing three-dimensional laser-scanned scenes of substations, which is a fast and suitable approach for substation modeling. The processing results of real point cloud data from substations demonstrate the applicability of this method in reconstructing point cloud scenes with large data volumes and complex noise. We employed the VCHC method to segment the entire substation point cloud scene, leveraging both the computational efficiency of voxel-based clustering methods and the noise resistance of density-based clustering methods, resulting in a segmentation accuracy exceeding 90%.

Building upon this, we adopted a classification approach that combines Dense SSD and ICP, achieving a classification accuracy of 92.86% for electrical equipment. Dense SSD accurately recognizes multiple device categories within fine-grained clusters, effectively avoiding inaccurate identification due to insufficient subdivisions. Moreover, ICP not only distinguishes different types of devices with subtle appearance differences but also accurately places the models into the substation scene.

## Data Availability

The datasets usedand/or analysed during the current study ara available from the corresponding author on reasonable request.
